# Clinical pharmacy services are reimbursed in Germany: challenges of real world implementation remain

**DOI:** 10.1007/s11096-022-01492-7

**Published:** 2022-11-17

**Authors:** Martin Schulz, Nina Griese-Mammen, Uta Müller

**Affiliations:** 1 Institute of Pharmacy, Freie Universität Berlin, Kelchstraße 31, 12169 Berlin, Germany; 2 Department of Medicine, ABDA – Federal Union of German Associations of Pharmacists, Heidestr. 7, 10557 Berlin, Germany

**Keywords:** Community pharmacy services, Medication review, Medication therapy management, Pharmaceutical services, Pharmacy

## Abstract

Over the last two decades, community pharmacy has experienced major changes as the role of pharmacists is evolving from a product to a service and a patient focus. As part of this change, new and innovative clinical pharmacy services aimed at improving medicines use and patient outcomes have been designed, both nationally and internationally. Since June 2022, five services are reimbursed by all statutory health insurance funds and private insurance companies in Germany: medication review for patients with polymedication; blood pressure control in hypertension; assuring proper inhalation techniques for patients receiving a new device or a device change; medication review including a follow-up for patients taking oral anticancer drugs or immunosuppressants post-transplantation. Beyond reimbursement, the upscaling and sustainable provision of these professional services are now the main challenges. Implementation research will provide important information for the further development of pharmaceutical care programs.

## Background

With a population of 83.3 million, Germany is the highest populated country in the European Union. Health insurance is mandatory, either through statutory or private health insurance. The statutory health insurance (SHI) system, consisting of nearly 100 funds covers 88% of the population i.e., approximately 73.3 million people.

The German primary care system consists of approximately 150,000 office-based physicians, and 18,256 community pharmacies (CPs), as of June 2022. In 2021, 53,300 of the approximately 68,800 pharmacists worked in CPs. Only pharmacists may own a pharmacy and a pharmacist may operate up to three subsidiaries nearby his/her main pharmacy. Therefore, no pharmacy chains exist. In 2021, 85.5% of the total community pharmacy turnover (59.93 billion euro without VAT) was dispensing fees for prescribed drugs.

In 2014, ABDA—Federal Union of German Associations of Pharmacists, the umbrella organization consisting of the 17 State Chambers of Pharmacists (all pharmacists) and the 17 State Associations of Pharmacists (community pharmacy owners only), released the policy paper “Pharmacy 2030—Perspectives on provision of pharmacy services in Germany”. This paper sees pharmacists maintaining a key role in primary care and improving and intensifying collaboration with other healthcare professions as well as actively shaping the healthcare network with clearly defined competencies, assuming responsibility for the safety and optimization of medication therapy and practices. In addition, current clinical pharmacy services (CPS) are outlined, alongside future prospects regarding the pharmacists’ role and range of CPS. There has been significant efforts to introduce CPS into standard care and practice. Training programs, guidelines, and working materials have been developed, and changes in the legislation to support this were achieved. Relevant milestones were:

- The introduction of clinical pharmacy in the academic curriculum for pharmacists in 2001.
- The definition of ‘medication management’ as a pharmaceutical service in 2012 in the pharmacy operating regulations (Ordinance on the Operation of a Pharmacy [ApBetrO Sect. 1a(3)6.]): “Medication management is a pharmaceutical activity, in which the entire medication of the patient, including self-medication, is repeatedly analyzed with the aim of improving medication safety and adherence by identifying and solving drug-related problems”.
- The development of advanced training concepts for medication review (MR) e.g., Apo-AMTS and ATHINA (since 2012).
- The development of a guideline for MR (2014, revision in 2017).
- A MR-curriculum of the Federal Chamber of Pharmacists (since 2015) [1].

For example, ATHINA is an educational program and has been offered by 11 regional chambers. The evaluation showed feasibility as well as an effect in terms of drug-related problems (DRP) having been identified and solved [2, 3]. As a limitation [4, 5], physicians are not involved as cooperating partners.

However, an interprofessional medication management program (MMP) was introduced in 2016 in which general practitioners (GPs) and CPs collaboratively performed MR and continuously followed-up on patients with medical and pharmaceutical tasks, respectively. This MMP has been implemented as part of ARMIN (“Arzneimittelinitiative Sachsen-Thüringen”), a project endorsed by the professional associations of statutory health physicians and community pharmacy owners and one SHI fund (AOK PLUS, 3.4 million insured persons) in two federal states, Saxony and Thuringia. Patients signing up for the program chose both a GP and a CP who jointly supervised the patients’ drug therapy. Specific tasks and responsibilities were assigned to each professional group. Integrating an electronic data transfer between GPs and CPs into the local software facilitated implementation [6]. The evaluation revealed that GPs and CPs shared most of the tasks in the MMP, as envisaged in the original concept, and many of their tasks complemented each other [7]. The evaluation of the primary outcomes of this MMP has been submitted for publication, recently.

In October 2020, after many years, even decades of research and negotiation, the German Federal Parliament adopted the “Law on Strengthening Local Community Pharmacies” (in German: *Gesetz zur Stärkung der Vor-Ort-Apotheken [VOASG]*) and legally stipulated the right of patients to CPS (in German: *pharmazeutische Dienstleistungen*). These are services that go beyond the legal obligation to counsel when dispensing drugs (Sect. 20 of the ApBetrO) and that improve the care of the patient. The pharmaceutical services include, in particular, measures by the pharmacies to improve medication effectiveness and safety (Sect. 129(5e) German Social Code Book V). All health insurance companies (statutory and private) were legally obligated to provide the pharmacies with a total of about 150 million euro per year for the provision of these CPS. The 150 million euro are available on top of the ‘normal’ reimbursement scheme for dispensing and counselling on prescribed drugs [8].

### Clinical pharmacy versus pharmaceutical care services

The European Society of Clinical Pharmacy (ESCP) defines that “Clinical Pharmacy aims to optimize the utilization of medicines through practice and research in order to achieve person-centered and public health goals. […].” This definition explicitly states that clinical pharmacy practice may be conducted regardless of the setting. The word “clinical” refers to the focus of clinical pharmacy activities—that is, patients rather than drugs, and not the setting in which they are provided [9].

The Pharmaceutical Care Network Europe (PCNE) defines that “Pharmaceutical Care is the pharmacist's contribution to the care of individuals in order to optimize medicines use and improve health outcomes [10]."

Therefore, the five newly reimbursed CPS below may also be called pharmaceutical care services.

### Reimbursed clinical pharmacy services

Since June 2022, after difficult negotiations with the National Association of SHI funds the following five CPS can be billed [8]:

**Medication review for patients with polymedication** in ambulatory care who are taking at least five systemic/inhaled drugs as long-term medication.

Medication reviews (MR) are a well-known strategy to improve medication safety and effectiveness. They can solve drug-related problems (DRP), improve medication appropriateness, guideline adherence, and clinical outcomes [2–5, 11–17]. Pharmacist-led MR programs in primary care settings have been commissioned in different countries over the past 20 years. Internationally, such services include Medicines Use Review (MUR) in the United Kingdom, MR in Denmark, MR provided by a clinical pharmacist consultant in Slovenia, Medication Therapy Management in the United States, MedsCheck in Canada, and Clinical Medication Review (CMR) and Home Medication Review (HMR) in Australia, among others [5, 11, 14, 18].

The newly reimbursed MR in Germany represents a type 2a MR [11, 12, 14–16] according to the PCNE definition [16]. Besides the medication history and the patient interview (‘brown bag review’: the patient brings all his/her medication (prescribed and OTC), in a ‘brown bag’), data sources such as medication schedules/plans, discharge letters, or doctors notes, these information are also considered if provided by the patient.

The PCNE recognizes three types of MR [16]. A type 2a MR is an intermediate MR that can be performed when the patient can be approached for information; clinical data are not a prerequisite (in a MR type 2b, there is no patient to speak to but clinical data are available [16]). Detected DRPs are evaluated and resolved as far as possible. For this purpose, consultation with the treating physicians can also take place with the patient’s consent. The primary care physician will receive a written report (also if the patient consents). We know from experience that patients usually provide their consent. Finally, the patient receives an updated medication schedule/plan listing his/her current entire medication.

A patient is entitled to one MR a year. In the event of significant changes (defined as at least three new or other systemic/inhaled drugs within four weeks as long-term medication), the service can be provided and invoiced again before the end of the 12 month period. The total duration of this MR is on average 80 min and is compensated at EUR 90 (+ VAT). Only pharmacists are authorized to provide this service. They must have completed an advanced training course based on the MR-curriculum of the Federal Chamber of Pharmacists. More than 10,000 pharmacists have already completed this course.

**Blood pressure control in hypertension**

Providing blood pressure (BP) control in hypertension [19–21] is directed at people with diagnosed hypertension who take at least one prescribed antihypertensive. This CPS can be invoiced for each patient—from two weeks after the start of therapy—once every 12 months. The service can be additionally invoiced in the event of a change in antihypertensive medication i.e., two weeks after the patient is presenting a prescription for a new/different antihypertensive (each EUR 11.20 + VAT).

According to guideline recommendations, the BP in the pharmacy should be taken after a 5-min resting period, with three BP measurements while seated with each measurement at intervals of one to two minutes. The BP values measured (including pulse rates) and the mean of the last two BP measurements are recorded on a guideline worksheet. If abnormally elevated BP values are measured, the pharmacy refers the patient to his/her GP [21]. This CPS may be provided by all pharmacy dispensing staff, including pharmacists and pharmacy technicians in training.

**Assuring proper inhalation techniques for patients receiving a new device or a device change**

Several national and international studies on the care of asthma and COPD patients delivered by community pharmacists have shown that pharmacist interventions help to improve patient outcomes such as quality of life, self-management skills, medication adherence and optimization of drug therapy [22–29]. The robust evidence has led to pharmacists’ involvement and recognition in the German National Disease Management Guidelines for Asthma and COPD, respectively [30]. In 2017, already more than 5,600 pharmacists had completed an advanced course of pharmaceutical care for asthma patients.

Patients from the age of six may now receive an offer to practice their inhalation technique in a quality-assured manner according to a standardized process [22–24, 30]. This is to improve the administration of inhaled drugs and to increase medication effectiveness and safety. This CPS may be provided by all pharmacy dispensing staff, but not by pharmacists and pharmacy technicians in training. It is invoiced at EUR 20 (+ VAT) and can be offered when a device is newly prescribed, when a device is changed, or if the patient has not received a practical training with their device in a doctor’s office or pharmacy in the past 12 months. In the latter case, the patient must also not be enrolled in a disease management program for asthma or COPD, according to self-reported information.

In addition, two other specific services have been introduced that affect smaller patient groups:

**Medication review with follow-up for patients taking oral anticancer drugs**

and

**Medication review with follow-up for patients taking immunosuppressants post-transplantation**

are consisting of the services described under the “Medication review for patients with polymedication”. Futhermore, the MR addresses specifics of oral anticancer therapy [31, 32] or immunosuppressive drug therapy after organ transplantation [33, 34]. As a follow-up, another consultation in the form of a semi-structured discussion takes place two to six months later, if necessary, in order to identify and solve possible problems with the new therapy and to strengthen adherence. These CPS can be offered once within the first six months after transplantation or after starting the oral anticancer therapy, and also in the case of a change in these specific drug classes. The remuneration is EUR 90 (+ VAT) for the MR. The second consultation, taking place two to six months after the initial MR is billable at EUR 17.55 (+ VAT). These two CPS may only be offered by pharmacists. They must have completed the same advanced training course based on the MR-curriculum, as mentioned above.

Currently, upscaling and sustainable provision of these CPS are the main challenges. ABDA provides comprehensive job aids such as interview guides, checklists, standard operating procedures, among others [35]. The 17 State Chambers of Pharmacists offer corresponding continuing education courses. In case of billing questions, the 17 State Associations of Pharmacists are responsible.

## Conclusions and outlook

Since June 2022, after over two decades of research and negotiation, five CPS provided by CPs are reimbursed by all SHI funds and private insurance companies in Germany. Continuously analyzing the claims data will provide robust data on the scale of the use. From the viewpoint of research, CPS applied in a real world mandate to investigate at least (a) the sustainability of implementation, exploring enablers and barriers, including workforce issues and (b) the added value of these remunerated services [7, 14, 22, 36–38]. The findings may also inform the development and implementation of CPS in other healthcare systems.
